# Osteohistological variation in growth marks and osteocyte lacunar density in a theropod dinosaur (Coelurosauria: Ornithomimidae)

**DOI:** 10.1186/s12862-014-0231-y

**Published:** 2014-11-25

**Authors:** Thomas M Cullen, David C Evans, Michael J Ryan, Philip J Currie, Yoshitsugu Kobayashi

**Affiliations:** Department of Ecology and Evolutionary Biology, University of Toronto, 25 Willcocks Street, Toronto, ON M5S 3B2 Canada; Department of Natural History, Royal Ontario Museum, 100 Queen’s Park, Toronto, ON M5S 2C6 Canada; Department of Vertebrate Paleontology, Cleveland Museum of Natural History, Cleveland, OH 44106-1767 U.S.A; Department of Biological Sciences, University of Alberta, Edmonton, AB T6G 2E9 Canada; Hokkaido University Museum, Hokkaido University, Sapporo, Hokkaido 060-0810 Japan

## Abstract

**Background:**

Osteohistological examinations of fossil vertebrates have utilized a number of proxies, such as counts and spacing of lines of arrested growth (LAGs) and osteocyte lacunar densities (OLD), in order to make inferences related to skeletochronology and mass-specific growth rates. However, many of these studies rely on samplings of isolated bones from single individuals. These analyses do not take individual variation into account, and as a result may lead to misleading inferences of the physiology of extinct organisms. This study uses a multi-element, multi-individual sampling of ornithomimid dinosaurs to test the amount of individual variation in the aforementioned osteohistological indicators. Based on these results we also assess the conclusions of previous studies that tested paleohistological hypotheses using isolated elements.

**Results:**

LAG number was found to be consistent within the hind limb bones of each individual, with the exception of the fibula, which preserves one additional LAG. Considerable differences in LAG spacing were found between elements of the sampled individuals, with larger variation found in elements of the foot compared with the femur, fibula, and tibia. Osteocyte lacunar density ranged between 29000 and 42000 osteocyte lacunae per mm^3^, and was found to vary more between hind limb bones of an individual and within bones, than between the average values of individuals.

**Conclusions:**

The variation between hind limb elements in LAG number and LAG spacing suggests that direct comparisons of these elements may be misleading, and that LAG spacing is not a reliable proxy for mass-specific growth rates of an individual. Sampling of multiple bones should be performed as an internal check of model-based LAG retro-calculation and growth equations. The observation that osteocyte lacunar density varies more between individual bone elements than between average individual values suggests that the choice of sampled element can greatly influence the result, and care should be taken to not bias interpretations of the physiology of fossil tetrapods.

**Electronic supplementary material:**

The online version of this article (doi:10.1186/s12862-014-0231-y) contains supplementary material, which is available to authorized users.

## Background

Osteohistological examinations of fossil vertebrates have provided important insights into their ontogeny and physiology [1-12]. In addition to the description of bone tissue types, considerable attention has been paid to skeletochronological indicators, such as the number and relative spacing of lines of arrested growth (LAGs) and other cyclical growth marks within the bone cortex [13-15]. The presence of tightly spaced growth marks within relatively avascular tissues in the outer margin of the bone cortex (the external fundamental system, or EFS, of some authors, e.g. Horner et al. [16], or the outer circumferential layer, or OCL, following Cormack [17]) has been used to infer somatic maturity in sampled individuals [7,14,15]. In the absence of an EFS, patterns in the relative spacing of LAGs has been used to infer the ontogenetic stage of an individual, in some cases using a single sectioned element [2,8,18-26]. On a finer scale, the density of osteocyte lacunae (osteocyte lacunar density, OLD) in the cortex of limb bones of tetrapods has been suggested to be related to mechanical forces and differential loadings, basal metabolic and growth rates, and element structural differences [27-29], and osteocyte lacuna morphology itself has also been shown to be related to these properties [27,29,30]. Recently, the densities of osteocyte lacunae have been used to predict basal metabolic rates and relative growth rates in fossil tetrapods, including dinosaurs [29].

Few studies to date have focused on variation in LAG spacing between different limb bones of a single individual; individual variation in osteocyte lacunar morphology [30,31] and density [27,28] in the long bones of tetrapods suggests that element choice and sampling location may influence studies using these histological parameters to infer life history traits in fossil taxa. Here, we describe bone microstructure and assess intra- and inter-skeletal variation in skeletochronological indicators (LAGs) and osteocyte lacunar density (OLD) in a sample from the Horseshoe Canyon Formation (Maastrichtian, Alberta) of ornithomimid theropod skeletons, three of which were found closely associated in the same stratigraphic horizon and likely represent individuals dervied from the same population [32]. Specifically, we use the intra-specific, multi-element osteohistological data to test two separate hypotheses: 1) that patterns of lines of arrested growth (LAG) spacing in different elements of a single individual provides a consistent signal for evaluating its relative maturity, and, 2) osteocyte lacunar densities (OLDs) are generally consistent between limb elements of a single individual, and between individuals of a given taxon due to their shared physiology and loading regimes. Growth marks, such as LAGs, are particularly well documented in theropod dinosaurs [5,7,18,25,33-36], and this detailed case study of individual variation in a single theropod taxon may provide insights that can be extended to other theropod dinosaurs, as well as tetrapods more generally.

## Results

### Histological description

**Figure 1:**
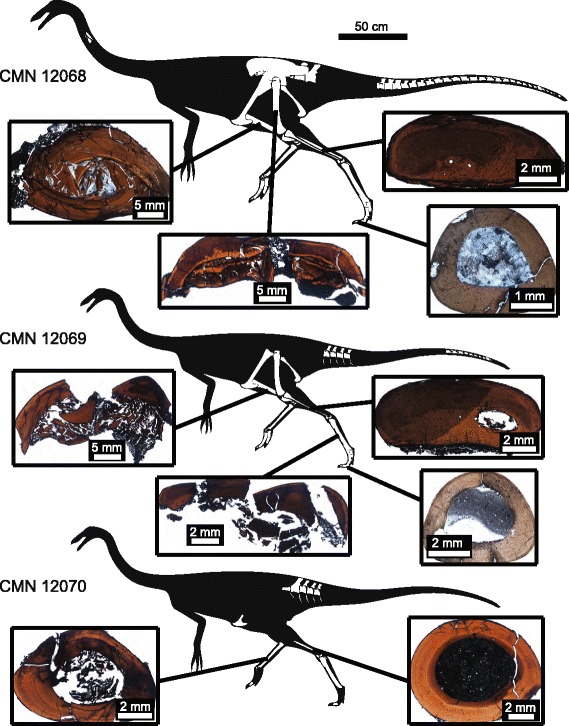
**Summary of sectioned elements of CMN 12068, CMN 12069, and CMN 12070.**

In each sampled element of the bonebed individuals (Figure 1), the bone matrix is consistent in being predominantly a woven-parallel complex, showing a combination of laminar, plexiform, and reticular patterns of vascularization. An external fundamental system (EFS) is absent in all elements, with all periosteal margins showing active primary bone deposition and well-vascularized tissue [10,16,17]. However, some differences exist in the relative distributions of vascularization and the degree of secondary remodelling in elements of each individual, and in the number of lines of arrested growth (LAGs) preserved.

**Figure 2:**
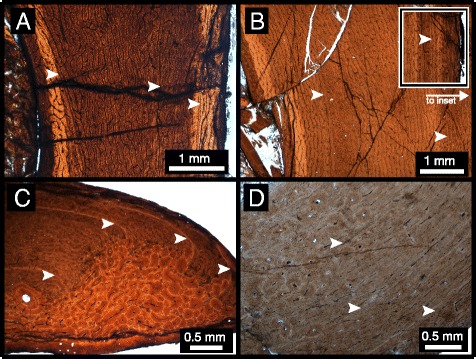
**Histological sections of CMN 12068. A**, femur; **B**, tibia; **C**, fibula; **D**, pedal phalanx. Arrows indicate LAGs.

The bone tissue of the femur of the largest individual (CMN 12068) (Figure 2A) ranges from laminar to plexiform, with the density of radial canals varying throughout the section. Secondary remodelling is minimal in most areas of the femur; however, there is a heavily remodelled section of the medial cortex that is associated with a slight external eminence of the periosteal margin where the cortex is thickened relative to the rest of the section. Three LAGs are visible in the femur, with the innermost LAG partially lost due to remodelling and cortical drift. The outermost LAG appears as a double LAG in some limited sections of the cortex. The tibia of CMN 12068 (Figure 2B) is comprised primarily of laminar bone, with some plexiform regions due to localized increases in the density of radial canals. Secondary remodelling occurs in the inner cortex, although secondary osteon development is not extensive, nor is there evidence of considerable outward expansion of the medullary cavity along the endosteal margin. Cortical thickness is inconsistent, with the medial side of the element being considerably thicker. Three LAGs that completely extend around the cortex are visible in the tibia. The fibula (Figure 2C) of this individual shows plexiform and reticular bone, with greater vascularization towards the periosteal margin. There is extensive secondary remodelling on the medial side of the cortex, including several large, well-developed, secondary osteons, and a poorly developed medullary cavity. Unlike the other sectioned elements of this individual, the fibula preserves four visible LAGs. The third pedal phalanx (Figure 2D) exhibits primarily laminar vascularization, with some regions appearing semi-plexiform to plexiform. There is secondary remodelling concentrated along the inner cortex, particularly along the ventral side of the phalanx. This area of the cortex is also slightly thicker than the lateral or dorsal sides. Like the femur and tibia, three LAGs are preserved in the phalanx.

**Figure 3:**
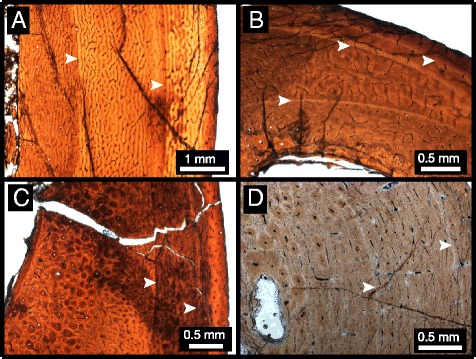
**Histological sections of CMN 12069. A**, tibia; **B**, fibula; **C**, metatarsal; **D**, pedal phalanx. Arrows indicate LAGs.

CMN 12069 (Figure 3A) is smaller than CMN 12068, but approximately the same size as CMN 12070. The tibia of CMN 12069 has primarily laminar to plexiform vascularization, with areas of reticular bone associated with increased density of radial canals. There is little to no secondary remodelling, nor is there evidence of erosion of the cortex via expansions of the medullary cavity. The cortex thickness is relatively consistent in the incompletely preserved section, and two LAGs are present. The fibula (Figure 3B) of this individual shows primarily reticular vascularization, with more extensive secondary remodelling on the medial side of the cortex. However, secondary remodelling is less extensive than in the larger fibula of CMN 12068. A well-defined medullary cavity is present, and the fibula preserves three LAGs. Metatarsal IV (Figure 3C) has reticular and plexiform vascularization, concentrated towards the inner cortex, and laminar vascularization concentrated towards the outer cortex. Much of the inner cortex is secondarily remodelled, although there are no outward expansions of the medullary cavity along the endosteal margin of the cortex. As in the tibia, but unlike the fibula, there are two LAGs present in the metatarsal. The pedal phalanx (Figure 3D) exhibits primarily laminar vascularization, with two LAGs present in the cortex.

**Figure 4:**
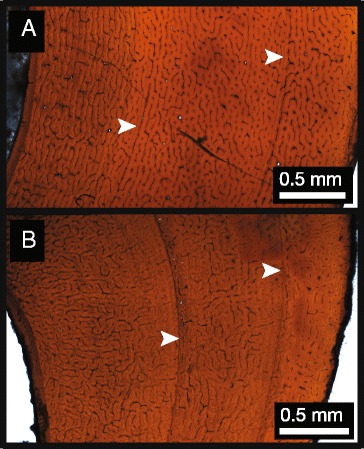
**Histological sections of CMN 12070. A**, metatarsal; **B**, tibia. Arrows indicate LAGs.

The tibia of CMN 12070 (Figure 4A) has woven-parallel complex bone tissue with primarily laminar to plexiform vascularization, with little to no secondary remodelling [10]. The cortex thickness appears relatively consistent, and two LAGs that extend around the cortex are visible. Like CMN 12069, the fourth metatarsal (Figure 4B) is comprised of plexiform and reticular vascularization concentrated towards the inner cortex, and laminar vascularization concentrated towards the outer cortex. There is secondary remodelling concentrated in the inner cortex and through sections of the medial and lateral outer cortex As in its associated tibia, there are two LAGs visible in the metatarsal of CMN 12070.

**Figure 5:**
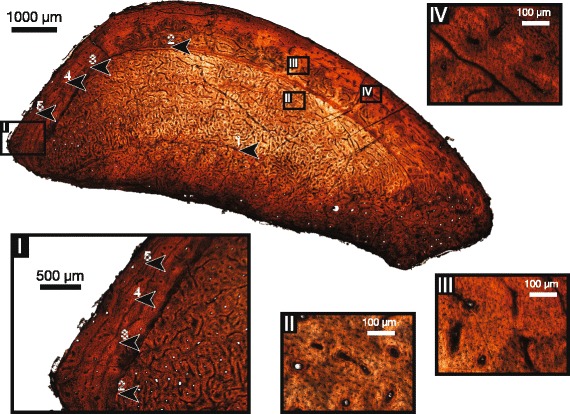
**Histological section of fibula of ROM 852.** Inset **(I)** shows outer cortex and detailed locations of LAGs 2–5. Inset **(II)** shows detail of inner cortex tissue and osteocyte lacunae. Inset **(III)** shows detail of middle cortex tissue and osteocyte lacunae. Inset **(IV)** shows detail of outer cortex tissue and osteocyte lacunae. Arrows indicate LAGs.

The fibula of ROM 852 (Figure 5) exhibits primarily plexiform vascularization with reticular vascularization in some sections towards the inner cortex, and less vascularized, more laminar tissue towards the periosteum. There is no definitive presence of an EFS/OCL. Secondary remodelling is relatively extensive on the medial side of the cortex. Five LAGs are present (indicated in Figure 5 and contained inset [I]), with inter-LAG spacing increasing between the first and second LAG, and decreasing considerably between the subsequent LAGs approaching the periosteum. As in CMN 12068, this specimen has double LAGs, most notably near the middle of the cortex in the second LAG, and additionally in variable positions of the fifth LAG. While most of the LAGs visibly extend around the cortex (the first and second most obviously, the fourth and fifth visible, but less distinct), the third LAG is visible in the posterior cortex, but becomes indistinct as it grades into the more heavily vascularized mid cortex and more remodelled anterior cortex.

### Variability in growth line spacing

**Figure 6:**
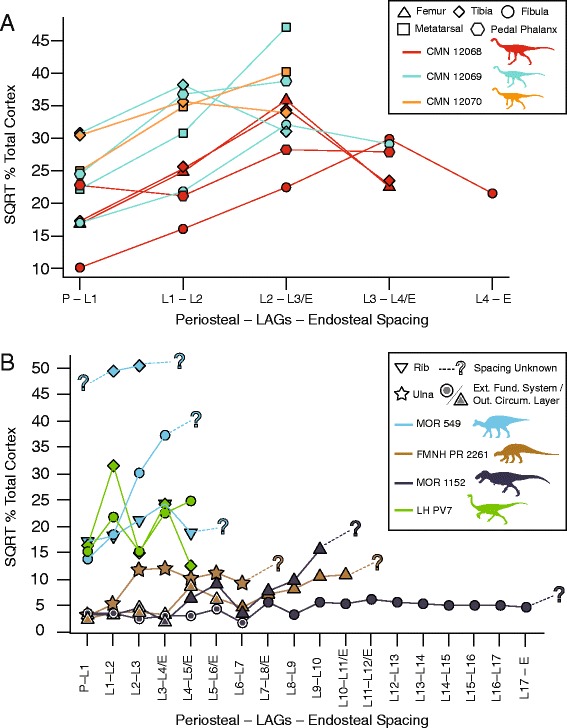
**Comparisons of LAG spacing in (A) sectioned ornithomimids CMN 12068, CMN 12069, and CMN 12070, and in (B) other published histological sections.** Abbreviations: SQRT, square root; LAG, line of arrested growth. Detailed legend shown in figure.

Among the three ornithomimids from the Horseshoe Canyon Formation bonebed (Figure 6A), there is a similarity in the pattern of LAG spacing in the femur, fibula, and tibia, with all three elements showing a relative increase in spacing from the periosteal margin to the innermost LAG. However, the lowermost hind limb elements (MT IV, pedal phalanx) did not show the same signal within a given individual; the metatarsals and pedal phalanges show a more consistent spacing between LAGs, and do not exhibit a decrease between the innermost LAG and endosteal margin. Although the absolute distance between LAGs varies within individual bones, the relative pattern of LAG spacing (increases versus decreases) within a single bone is consistent between different measurement transects (Figure 5). However, the relative proportion of cortex defined by each cycle can vary in different measurement transects.

### Osteocyte lacunar density

Osteocyte lacunar density (OLD) in the total sample of four ornithomimids from the Horseshoe Canyon Formation ranged from ~29000 to ~42800 osteocyte lacunae (OL) per mm^3^; standard deviations (SD) ranged from ~600 to ~6000 OL/mm^3^, depending on the individual or element sampled (Table 1). The individual specimens averaged 34955 OL/mm^3^ (SD of 1855 OL/mm^3^) for CMN 12070, 35002 OL/mm^3^ (SD of 5943 OL/mm^3^) for CMN 12069, 37176 OL/mm^3^ (SD of 2982 OL/mm^3^) for CMN 12068, and 37314 OL/mm^3^ (SD of 1408 OL/mm^3^) for ROM 852; the global individual average OLD is ~36112 OL/mm^3^ (SD of 1310 OL/mm^3^). Sampling of the medial and lateral sides of each sectioned bone of CMN 12068 yielded 38681 OL/mm^3^ and 41533 OL/mm^3^ for the femur, (mean of 40107 OL/mm^3^, SD of 2017 OL/mm^3^) 34052 OL/mm^3^ and 33180 OL/mm^3^ for the tibia, (mean of 33616 OL/mm^3^, SD of 617 OL/mm^3^) 36840 OL/mm^3^ and 40000 OL/mm^3^ for the fibula, (mean of 38420 OL/mm^3^, SD of 2234 OL/mm^3^) and 34769 OL/mm^3^ and 38354 OL/mm^3^ for the pedal phalanx (mean of 33561 OL/mm^3^, SD of 2535 OL/mm^3^). Sampling of the inner, middle, and outer cortex of the fibula of ROM 852, yielded results of ~36004 OL/mm^3^ (SD of 1239 OL/mm^3^), ~37137 OL/mm^3^ (SD of 1202 OL/mm^3^), and ~38802 OL/mm^3^ (SD of 1508 OL/mm^3^) respectively.

**Table 1:** **Osteocyte lacunar density (OLD) of sampled ornithomimid specimens**

**Specimen & sampling location in cortex**	**Field of view (FOV) dim (L) (μm)**	**Field of view (FOV) dim (W) (μm)**	**Field of view (FOV) thickness s (μm)**	**FOV in μm^3**	**FOV in mm^3**	**# OL in FOV**	**OLD (#/mm^3)**	**Bone avg. OLD**	**Bone avg. OLD std. dev.**	**Individual avg. OLD**	**Individual avg. OLD std. dev.**	**GLOBAL AVG OLD**	**GLOBAL avg. OLD std. dev.**
CMN 12070 tibia	250	250	60.0	3750000	0.00375	136	36267	36267	-				
CMN 12070 mtIV	250	250	83.7	5231250	0.00523	176	33644	33644	-	34955	1855		
CMN 12069 tibia	250	250	60.0	3750000	0.00375	136	36267	36267	-				
CMN 12069 fibula	250	250	53.0	3312500	0.00331	142	42868	42868	-				
CMN 12069 mtIV	250	250	73.0	4562500	0.00456	136	29808	29808	-	35002	5943		
CMN 12069 ped. phal.	250	250	68.5	4281250	0.00428	133	31066	31066	-				
CMN 12068 femur medial	250	250	63.7	3981250	0.00398	1154	38681						
CMN 12068 femur lateral	250	250	57.4	3587500	0.00359	149	41533	40107	2017				
CMN 12068 tibia medial	250	250	68.6	4287500	0.00429	146	34052						
CMN 12068 tibia lateral	250	250	65.1	4068750	0.00407	135	33180	33616	617	37176	2982		
CMN 12068 fib medial	250	250	59.5	3718750	0.00372	137	36800						
CMN 12068 fib lateral	250	250	56.0	3500000	0.00350	140	40000	38420	2234				
CMN 12068 ped. phal. medial	250	250	49.7	3106250	0.00311	108	34769						
CMN 12068 ped. phal. lateral	250	250	41.3	2581250	0.00258	99	38354	36561	2535				
ROM 852 fibula inner 1	250	250	52.0	3250000	0.00325	115	35385					36112	1310
ROM 852 fibula inner 2	250	250	46.0	2875000	0.00288	104	36174						
ROM 852 fibula inner 3	250	250	49.0	3062500	0.00306	109	35592	36004	1239				
ROM 852 fibula inner 4	250	250	45.0	2812500	0.00281	107	38044						
ROM 852 fibula inner 5	250	250	51.0	3187500	0.00319	111	34824						
ROM 852 fibula middle 1	250	250	54.0	3375000	0.00338	128	37926						
ROM 852 fibula middle 2	250	250	48.3	3018750	0.00302	111	36770						
ROM 852 fibula middle 3	250	250	47.8	2987500	0.00299	115	38494	37137	1202	37314	1408		
ROM 852 fibula middle 4	250	250	50.4	3150000	0.00315	117	37143						
ROM 852 fibula middle 5	250	250	52.5	3281250	0.00328	116	35352						
ROM 852 fibula outer 1	250	250	55.0	3437500	0.00344	137	39855						
ROM 852 fibula outer 2	250	250	53.2	3325000	0.00333	124	37293						
ROM 852 fibula outer 3	250	250	50.4	3150000	0.00315	125	39683	38802	1508				
ROM 852 fibula outer 4	250	250	55.3	3456250	0.00346	128	37034						
ROM 852 fibula outer 5	250	250	54.6	3412500	0.00341	137	40147						

**Figure 7:**
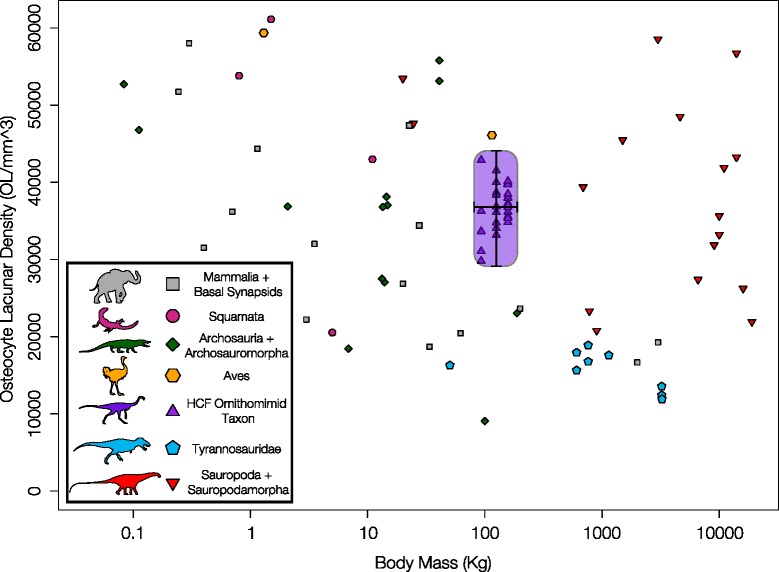
**Range of intra- and inter-individual variation in osteocyte lacunar density (OLD) in Horseshoe Canyon Formation (HCF) ornithomimid taxon compared with point-sampling of species from Stein & Werner** **[** 29 **].** Mammal data from Stein & Werner 2013 primarily derived from Bromage et al. [44]. Purple box indicates range of measured variation in Horseshoe Canyon Formation Ornithomimid taxon.

**Figure 8:**
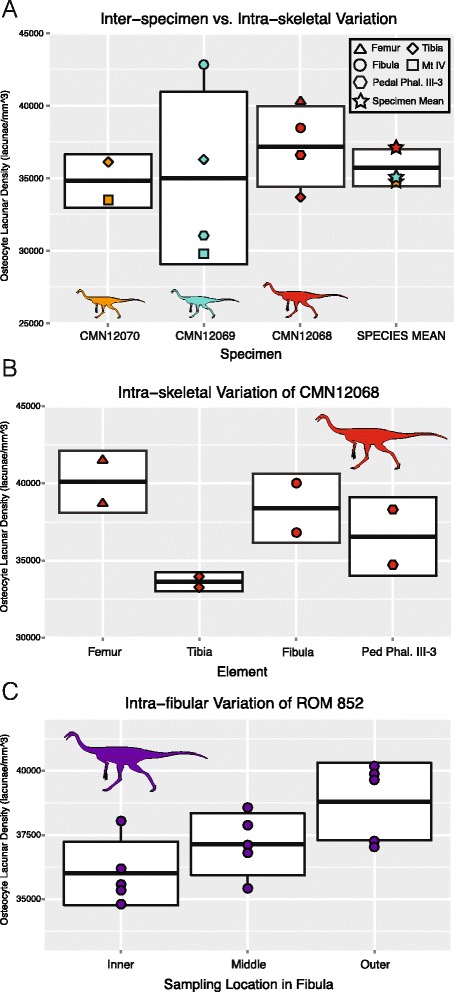
**Variation present in osteocyte lacunar density (OLD) at different sampling scales. A)** intra-skeletal vs. inter-specimen (species mean). **B)** intra-skeletal variation of CMN 12068 showing mean for each element, as well as lateral and medial values for each sampled element. **C)** intra-element variation present across a transect of the inner-middle-outer cortex of the fibula of ROM 852, showing the effect of ontogeny on OLD values. Each box represents the standard deviation around the mean (solid black line) of each sampling unit (species, individual, element, or location within element). Each OLD measurement is indicated by a symbol indicating sampling location. Detailed legend shown in figure.

For an estimated ornithomimid body size range of 90–125 kg [37], the minimum and maximum, average OLD values of the Horseshoe Canyon Formation ornithomimids plot midway between birds/mammals and non-avian theropod dinosaurs [29], but show considerable variation when individual bone measurements are plotted (Figure 7). This variation is illustrated further through a comparison of the range of OLD values for each element of the three bonebed individuals against the mean OLD values for the species derived from the individual averages (Figure 8A), a comparison of the variation in OLD within (via medial and lateral sampling) and between elements of CMN 12068 (Figure 8B), and a detailed comparison of the OLD values across a transect through the inner, middle, and outer cortex of the fibula of ROM 852 (Figure 8C). The latter comparison was also assessed via a series of ANOVAs, which found no significant differences in the OLD between the inner and middle cortex (p = 0.79), between the inner and outer cortex (p = 0.06), or between the middle and outer cortex (p = 0.97).

## Discussion

### Comparative osteohistology

All examined ornithomimid sections are characterized by woven-parallel complex tissue, with a range of laminar to reticular vascularization (primarily plexiform), and lack evidence of an EFS/OCL [10]. Each element sectioned has at least two LAGs. Within each bonebed individual, there is consistency in the number of LAGs within each element, with the exception of the fibula, which consistently has one more LAG. Additionally, localized double LAGs are present in the femur of CMN 12068 and the fibula of ROM 852, which suggests differential growth and localized suspension of bone deposition [19]. The femur of CMN 12068, the fibulae of CMN 12068, CMN 12069, and ROM 852, as well as the fourth metatarsal of CMN 12070, show localized secondary remodelling across the cortex that is likely related to adductor muscle attachment [23]. CMN 12069 and CMN 12070, the smaller individuals within the sample (metatarsal length ~29.3 cm & ~29.0 cm, respectively), show consistency in the number of LAGs in corresponding elements, which supports the hypothesis that this bonebed represents a mass death assemblage where the preserved individuals are part of the same cohort [32]. In the largest bonebed individual, all corresponding elements preserve one additional LAG compared to those of the smaller individuals, suggesting that it is at least one year older [7,11,21,38]. ROM 852, the largest individual overall (Additional file 1), had one more LAG in the fibula than CMN 12068. Although there is no evidence of an EFS/OCL in ROM 852, there is a decrease in tissue vascularity in the outermost cortex that suggests a reduction in bone growth rate [6,39]. As no EFS/OCL is preserved in any of the sectioned ornithomimid elements from this study, none of these individuals had yet reached skeletal maturity, and were likely still growing in body size. These specimens are of similar overall size to many other known North American ornithomimids (Additional file 1), including TMP 1995.110.1, which is reported to have an EFS/OCL [40]. This suggests that the maximum size of North American ornithomimids is not yet fully understood, and may show a large degree of individual or taxonomic variation.

This multi-element, multiple individual sampling approach permits the first in-depth, quantitative assessment of individual variation in osteohistological traits of a theropod dinosaur species. Differences in LAG counts in bones from the same individual is a pattern observed in other dinosaur histology studies [7,13,16,41], and is not unexpected due the possibility of different rates of bone growth and remodelling in different parts of the skeleton. However, in this study of hind limb elements, only the fibula differs from the other sampled elements in that it preserves one additional LAG among the multiple ornithomimid individuals. This differs from other studies that have sampled a wider representation of bones within the skeleton and have shown more variation within multiple elements of single individual [7,16,39,42]. The presence of an additional LAG in the fibula suggests that it may be a preferred element for skeletochronology in theropods, because it preserves the longest growth record due to a small medullary cavity and less remodelling, as suggested in previous studies [4,13]. A potential drawback of using the fibula is that its irregular shape, coupled with strong allometric growth in diaphysis shape (e.g., Figure 5), complicates retro-calculation-based age reconstructions and growth-curve model-fitting. Femora and tibiae do not have these growth related problems thus making them more favourable elements in equation-based retro-calculation growth reconstructions [38]. Therefore, the diaphysis of these primary weight-bearing bones may be more effective for reconstructing growth curves than fibulae, despite the fact that the fibula appears to consistently preserve more LAGs in theropods. Therefore, we propose that a within-individual, multi-element sampling approach can act as an internal check of retro-calculation-based age estimations, and that this will be the most powerful means of reconstructing growth curves of extinct vertebrates going forward.

### LAG spacing

Spacing between lines of arrested growth is often used to qualitatively assess the relative growth rates and maturity in isolated bones, e.g. *Tyrannosaurus* [5,7], *Alioramus* [18], *Raptorex* [23,25,26], and a putative Alaskan ornithomimosaur [26]. This method assumes that a decrease in LAG spacing from the inner cortex to the periosteum corresponds with an individual approaching somatic maturity. In our sample, LAG spacing is variable between different elements within a single individual (Figure 6A). The femur, tibia, and fibula do show a similar trend of decreasing distance between LAGs, regardless of where within the cortex the spacing is measured. However, the metatarsal and pedal phalanges do not show the same pattern as their corresponding upper limb bones. Here, distances between LAGs in the outer cortex, which presumably represent the same periods of growth as record in the crus and femur, are more variable and do not show constant decreases in spacing towards the periosteal surface. Therefore, hind limb and foot bones of a single individual would yield different assessments of relative maturity if analysed in isolation. For example, the inference that an Alaskan ornithomimosaur [26] was approaching somatic maturity at the time of death based on a decreasing pattern of LAG spacing in a single, isolated metatarsal, is poorly substantiated.

This type of variability in LAG spacing between elements of a single individual is also evident in other fossil studies (Figure 6B). Differing LAG spacing signals also appear to be present within different elements of an individual adult *Hypacrosaurus stebingeri* (MOR 549), although the presence of an EFS/OCL makes this difficult to show definitively; the fibula shows a pronounced increase in LAG spacing from the periosteal margin to the inner cortex, whereas the rib has more variable spacing between LAGs. In addition, alternating patterns of increase vs. decrease in LAG spacing through the cortex are also present in both the tibia and fibula of the Asian ornithomimid *Sinornithomimus dongi* [11], as well as in a femur of *Tenontosaurus* [12]. Variability in LAG spacing from the inner cortex to the periosteum is consistent with recent extant experimental work that shows that different long bones of an individual grow at different rates [22], and may also relate to annual variation in resource availiblity [9,20], or functional responses to ontogenetic gait shifts [43], among other factors. These problems could be further compounded if spacing is not assessed at comparable locations in the cortex, as they could lead to different interpretations of growth or ontogenetic status (such as in *Raptorex*, which has been suggested to represent either a small-bodied nearly-mature animal [25] or a juvenile *Tarbosaurus* [23], with both arguments made partly using LAG spacing). This demonstrates the unreliability of simple LAG spacing patterns for inferring a particular ontogenetic stage or relative maturity across a wide range of taxa or elements in somatically immature specimens where an EFS/OCL is not present [7,16].

### Osteocyte lacunar density

Recently, Stein and Werner [29] quantified and compared osteocyte lacunar density (OLD) in a sample of adult limb elements from a variety of extant and extinct taxa, which included mammal OLD data from a study by Bromage et al. [44]. They found significant relationships between OLD and body mass, and body mass-specific OLD and relative growth rates. Whereas the Stein and Werner study did not explicitly address how intra- and/or inter-individual variation in OLD may limit the interpretive power of their results, care was taken to sample from the outer cortex of each element, sample the femur when available (68% of sampled elements in the sample), and to use the largest individual per taxon in regression analyses when more than one sample was taken (as opposed to a mean taxon value), in order to avoid variation in OLD values resulting from ontogeny or sampling location. Although 41 percent of taxa included in Stein and Werner [29] were represented by more than one individual or sample (e.g., cf. *Trilophosaurus*, *Hyperodapedon*, *Rhamphorhynchus*, *Albertosaurus*, and *Tyrannosaurus*, in which more than two individuals were sampled), intra-specific, intra-individual, and intra-element variation in OLD was not explicitly quantified or discussed in context of their results. Given the variation in lacunar morphology (size and shape) between elements [30,31,45], variation in OLD within individual human femora [27] and between fore-limb elements of modern taxa [28], as well as quantification issues to due sectioning plane [45], intra-specific variation is potentially problematic when trying to predict aspects of organismal palaeobiology using osteocyte lacunar density.

Measured ornithomimid osteocyte lacunar densities varied within individual elements, and between elements of the same individual (Table 1). All OLD values calculated for each individual are larger than those of larger tyrannosaurid theropods sampled by Stein and Werner [29], as predicted by the inverse relationship between OLD and body mass. OLD values (all raw counts and global average) are relatively larger than similarly sized mammals, but plot between mammals and birds of similar body size [29] (Figure 7). This result is not unexpected, given the status of ornithomimids as small- to medium-bodied theropods [46,47]. However, our analysis indicates that there is considerably more variation in OLD between elements of an individual than in different positions within a single element (Figure 8B&C), or in the average OLD between different individuals (Figure 8A). This level of variation between elements of single individuals suggests that estimating metabolic [28,29] or genome size [31] characteristics of extinct organisms may be more complex than previously considered, and OLD counts may be affected by individual variation and allometry of different skeletal elements, as noted in studies of extant mammals [27-29,44]. Despite this high intra-skeletal variation, the mean OLDs of each specimen, calculated from the lacunar densities of multiple sampled elements in its skeleton, are very similar, despite each specimen not always being sampled from the same set of elements. Given this consistency, multi-element sampling and the use of individual/species mean OLDs is recommended in order to capture and control for the high variation present in sampling isolated individual elements.

Stein and Werner [29] postulated a relationship between ontogenetic stage and OLD, and suggested sampling of OLD in the outer cortex of mature animals to control for changing OLD throughout ontogeny. Our data suggest that, when sampling element and location is kept consistent, ontogenetic variation in OLD appears negligible. Over the ontogenetic size range investigated here (Figure 8C), no significant differences were found in sampling sites in the inner, middle, or outer cortex. However, it should be noted that none of our sampled series represents a skeletally-mature animal, given the lack of EFS/OCL, and there appeared to be a weak, albeit non-significant, positive trend towards increasing OLD through growth.

## Conclusions

Histological thin sectioning of multiple hind limb elements of three semi-articulated ornithomimid individuals, and a fibular thin section from a larger, stratigraphically contemporaneous specimen of *Ornithomimus edmontonicus* show a consistent pattern in LAG numbers. Each sectioned element within an individual has the same LAG number, with the exception of the fibula, which possesses one additional LAG. None of the sampled individuals appear to be skeletally mature, despite being of similar size to previously published mature individuals, suggesting a greater degree of individual variation in growth within North American ornithomimids than has been previously reported. Spacing of LAGs shows two distinct patterns, with femur/tibia/fibula LAG spacing decreasing relatively consistently from the inner to outer cortex, while the metatarsal/phalanx spacing remains more variable in spacing throughout the cortex. LAG spacing appears to be variable, and too easily affected by other factors of bone growth and modification, to be of repeatable use in assessing growth throughout ontogeny of individual animals or species (not counting the presence of an EFS/OCL). Similarly, osteocyte lacunar density appears more highly variable when making comparisons between bones of a single individual than when comparing the average values of different individuals. This suggests that, at least within closely related groups, element choice may have a greater impact on the measured result than ontogenetic stage of the individual. Localized factors relating to differential limb growth may play a larger role in the distribution and density of osteocyte lacunae than previously thought, and are potentially biasing the interpretations of analyses based on single samplings of isolated specimens.

## Methods

Three articulated partial ornithomimid skeletons from the Horseshoe Canyon Formation (HCF) were histologically sampled with permission from the Canadian Museum of Nature (CMN) for this study. The skeletal completeness, anatomy, and taphonomy of CMN 12068, 12069, and 12070 (cf. *Ornithomimus edmontonicus*, originally *Dromiceiomimus brevitertius*) was described by Cullen et al. [32]. One additional specimen sampled from the HCF was Royal Ontario Museum (ROM) 852 (*O. edmontonicus*, originally *Struthiomimus ingens*). The elements sampled were: CMN 12068 (femur, tibia, fibula, phalanx III-3), CMN 12069 (tibia, fibula, metatarsal IV, phalanx III-3), CMN 12070 (tibia, metatarsal IV), ROM 852 (fibula).

All thin-sections were created at the Royal Ontario Museum. Samples were initially embedded in Castolite AP polymer resin and cut as transverse diaphyseal sections using a Buehler Isomet 1000 wafer blade low-speed saw. These sections were then mounted to glass (CMN 12068, 12069, 12070) or plexiglass (ROM 852) slides using Scotch-Weld SF-100 cyanoacrylate and ground down to approximately 50–80 μm thick (for specific section thicknesses, see Table 1) using a Hilquist grinding cup, being ground progressively finer by hand using silicon carbide powder. Thin-sections were examined and photographed under plane-polarized light using a Nikon DS-Fi1 camera mounted to a Nikon AZ-100 microscope fitted with cross-polarizing and lambda filters. Specimen images were processed using Nikon NIS-Elements (Basic Research) v. 3.13.

Spacing between LAGs (=zone thickness) was measured in micrometres for each sectioned element of CMN 12068, CMN 12069, and CMN 12070. Measurements of LAG spacing were taken from the periosteal margin to the first visible LAG, then between each LAG, and from the innermost visible LAG to the endosteal margin of the cortex. Measurements were taken where the cortex was the thickest without extensive remodelling, or in the case of the tibia and metatarsal of CMN 12069, where the section of the cortex was most complete. This was done in an attempt to maximize comparability between sections, while minimizing subjectivity in space differences due to intra-element allometry. These values were then converted into percentages of the total cortex to allow for comparisons of relative spacing between elements. This was repeated for previously published histological sections of *Hypacrosaurus* [16], *Tenontosaurus* [12], *Sinornithomimus* [11], *Tyrannosaurus* [7], and *Raptorex* [23,25] and plotted in Figure 6B.

Osteocyte lacunar densities (OLD) were measured following the method (with slight modification) of [29]. Sections were re-imaged at approximately 32× magnification, and a 250 μm × 250 μm area of the outer cortex was selected from the resulting images. Images were taken at each focal plane of this square and combined into a single image via z-stacking. In the selected area of this z-stacked image, osteocyte lacunae of each element were counted three times and averaged. The lacunae counts were then converted to lacunar density per 1 mm^3^ of cortex (Table 1). Additional density counts per individual were obtained through averaging of individual element values. Each sectioned bone of CMN 12068 was resampled at locations on opposite sides of the outer cortex to additionally assess variation within an individual element. Osteocyte lacunar densities of ROM 852 were obtained from positions in the inner, middle, and outer bone cortex. Each position was sampled five times, and the inner/middle/outer measurements were compared using ANOVA in the ‘stats’ package in R [48]. Osteocyte lacunar measurements were plotted with those of Stein & Werner [29] in order to further illustrate the range of individual and inter-element variation in a single taxon of theropod (Figure 7), with the body mass estimated for the largest ornithomimid specimen (ROM 852) using the ‘MASSTIMATE’ package in R and the methods described in Campione et al. [37], with body masses of the other sub-adult individuals estimated using Developmental Mass Extrapolation [41] based on known/estimated femur lengths (Additional file 1). Osteocyte lacunar densities for sample, and the resulting means and standard deviations were also plotted as box plots in Figure 8, using the ‘ggplot2’ package in R [49].

### Availability of supporting data

Full-section images and images used for OLD calculations are available in the MorphoBank repository, project 1218 at http://morphobank.org.

## Additional file

Additional file 1:
**Comparative limb lengths of North American ornithomimids.**
